# Oncogenic Wnt3a: A Candidate Specific Marker and Novel Molecular Target for Hepatocellular Carcinoma

**DOI:** 10.7150/jca.31599

**Published:** 2019-10-08

**Authors:** Wenjie Zheng, Min Yao, Miao Fang, Liuhong Pan, Li Wang, Junling Yang, Zhizhen Dong, Dengfu Yao

**Affiliations:** 1 Research Center of Clinical Medicine, Affiliated Hospital of Nantong University, Nantong 226001, Jiangsu Province, China;; 2 Department of Medical Immunology, Medical School of Nantong University, Nantong 226001, Jiangsu Province, China;; 3 Department of Medical Informatics, Medical School of Nantong University, Nantong 226001, Jiangsu Province, China;; 4 Department of Diagnostics, Affiliated Hospital of Nantong University, Nantong 226001, Jiangsu Province, China.

**Keywords:** Hepatocellular carcinoma, Wnt3a, Specific diagnosis, Target therapy, Biomarker

## Abstract

**Background and aim:** It is of the utmost importance for the specific diagnosis and effective therapy of hepatocellular carcinoma (HCC). Abnormality of oncogenic Wingless-type MMTV integration site family member 3a (Wnt3a) has been associated with progression of HCC. In this study, we aimed to evaluate Wnt3a as a novel biomarker and target for HCC.

**Methods:** Circulating Wnt3a levels were quantitatively detected in a cohort of chronic liver diseases by an enzyme-linked immune-absorbent assay (ELISA). Hepatic Wnt3a expression in HCC and para-cancerous tissues was analyzed by immunohistochemistry. Prognostic value of Wnt3a for HCC was discovered in the cohort from the Cancer Genome Atlas (TCGA). Dynamic alterations of Wnt3a levels were detected in the hepatocarcinogenesis model induced by 2-acetylaminofluorene. Effects of Wnt3a on biological behaviors were evaluated *in vitro* and *in vivo* based on Crispr/Cas9.

**Results:** Up-regulated Wnt3a levels were observed in serum of HCC patients with high specificity and sensitivity for HCC diagnosis. Combination of Wnt3a and AFP could improve sensitivity to 93.9% in serological detection. In addition, Wnt3a expression in HCC tissues was significantly higher than that in para-cancerous tissues. The cohort of TCGA demonstrated that high Wnt3a expression led to a poor survival of HCC patients, especially in cases at advanced stages. Furthermore, the hepatocarcinogenesis model showed that Wnt3a dynamically increased in the development of HCC. Functionally, silencing Wnt3a by Crispr/Cas9 suppressed the proliferation, colony formation, induced cell cycle arrest of HCC cells by de-activating Wnt/β-catenin pathway *in vitro*, and inhibited xenograft tumor growth* in vivo*.

**Conclusions:** Oncogenic Wnt3a could be considered as a candidate biomarker and novel target for HCC.

## Introduction

Hepatocellular carcinoma (HCC) is still one of the most common cancers with leading cause of cancer in the world^1^, particularly in the inshore area of the Yangtze River^2^. The main etiological factors of HCC include chronic hepatitis B virus (HBV) or hepatitis C virus (HCV) infection, aflatoxin B1, and non-alcohol fat liver diseases (NAFLD)^3^. Chronic HBV carriers have a 5 ~ 15-fold increased risk of HCC compared with the general population, partially due to the activation of cellular Wnt/β-catenin and transforming growth factor-β (TGF-β) pathways^4^, ^5^. Early discovery and effective treatments are of the utmost importance for HCC therapy and prognosis. Serological biomarkers are widely used in the early diagnosis of HCC with the features of non-invasive, accuracy, and flexibility^6^. Despite of the wide application of alpha-fetoprotein (AFP), its false-negative rate may be as high as 40 % for HCC patients at early stage^7^. To date, some novel biomarkers including circulating glypican-3 (GPC-3)^8^ and microRNAs^9^ have been proposed. However, the overall efficiency is still unsatisfactory, especially in HCC patients with low AFP level or small-size tumor.

Accumulating evidence demonstrate that Wnt/β-catenin signaling molecules play significant roles in physiology, consisting by total 19 highly conserved secreted cysteine-rich glycoproteins with distinct functions. For instance, Wingless-type MMTV integration site family member 1 (Wnt1), Wnt2, Wnt3, Wnt3a, Wnt8a, Wnt8b, Wnt10a, and Wnt10b activate the canonical Wnt signaling and promote tumor progression, while Wnt4, Wnt5a, Wnt5b, Wnt6, Wnt7a, Wnt7b, and Wnt11 stimulate the non-canonical Wnt pathway as tumor suppressors^10^. The alterations of the Wnt/β-catenin signaling in pathology were related to numerous genetic/epigenetic abnormalities affecting cellular persistence, multiplication, migration, alteration and genomic instability^11^. Recently, abnormal expression of the Wnt signaling was reported closely associated with the occurrence, progression, and prognosis of HCC^12^.

Wnt3a is one of the most well-known Wnt ligands due to its critical roles in embryonic development as a pivotal component of the mesoderm gene. However, Wnt3a has recently been considered as an oncogenic factor in cancers of colon, breast, lung, and esophageal squamous cell^13^. Notably, silencing Wnt3a could inhibit proliferation, invasion, and chemo-resistance in glioma derived stem-like cells^14^. Previously, we first discovered the abnormal expression of Wnt3a in sera and cancerous tissues of HCC patients^15^. Nevertheless, the exact underlying mechanisms of Wnt3a and its alteration during malignant transformation of hepatocytes still remain to be explored. Therefore, this study aimed to further investigate the role of Wnt3a in clinical value and molecular-target for HCC.

## Methods

### Serum samples

Total 400 patients with benign or malignant liver diseases from the Affiliated Hospital of Nantong University, China between Jan. 2012 and Jan. 2017 were enrolled in this study. They were divided into four groups: HCC (n = 180), liver cirrhosis (LC, n = 80), chronic hepatitis (CH, n = 80), and NAFLD (n = 60). HCC was diagnosed according to the diagnostic criteria set by the 2012 Chinese National Collaborative Cancer Research Group. Among the 180 HCC (135 males, 45 females; ages ranged 35-86 years old) cohort, no patients received any pretreatment and all cases were confirmed by pathological examination. 59 cases with AFP level more than 20 ng/ml; 84 cases with lymph node metastasis; 47 cases with postoperative recurrence, 121 cases with positive hepatitis B surface antigen (HBsAg), and 81 cases with tumor size less than 3.0 cm. Serum AFP exceeding 20 μg/L was considered positive. Written informed consents were provided from all patients. Healthy persons with hepatitis B markers (HBsAg, HBcAb, and HBV-DNA) negative and normal serum alanine aminotransferase (ALT) levels as normal controls (NC, n = 80) were obtained from the Nantong Central Blood Bank in China. The study was in accordance with the Declaration of Helsinki and approved by the Ethics Committee of Affiliated Hospital of Nantong University, China.

### Liver tissue samples

Human liver cancerous and their surrounding tissues in this study were obtained from 60 HCC patients who underwent operations between Jan 2012 and Aug 2017 at the Affiliated Hospital of Nantong University, China. All sections were reviewed for confirmation of the original pathological and clinical diagnosis. Related clinical information including gender, age, tumor number, cirrhosis, HBV, AFP, and periportal cancer embolus were collected from their medical records. Tumor staging was based on the 6th edition of tumor-node-metastasis (TNM) classification of the International Union Anti-Cancer. This study was approved by the Ethics Committee of Affiliated Hospital of Nantong University, China (TDFY2013008), and in accordance with World Medical Association Declaration of Helsinki. Written informed consent was obtained from patients who participated in this study. The survival analysis was conducted by the Kaplan-Meier plotter, which was built based on the data of Cancer Genome Atlas (TCGA).

### Hepatocarcinogenesis model

Rat hepatocarcinogenesis model was made according to the previous method^16^. Male Sprague-Dawley rats (4-wk-old, body weight 140-150 g) were supplied by the Animal Center of Nantong University. Rats were maintained under pathogen-free conditions with a 12-h light-dark cycle. Malignant transformation of rat hepatocytes was induced by 2-acetylaminofluorene (2-AAF; Sigma). Sera and livers of rats were collected from rats sacrificed biweekly for immunochemistry and specific concentration of Wnt3a. All animal procedures were approved by the Animal Care and Use Committee of Nantong University, China.

### Cell culture

Human HepG2 cells were obtained from American Type Culture Collection (ATCC, USA) and grown in Dulbecco's modified Eagle's medium (DMEM, Hyclone, USA) containing 10 % fetal bovine serum (FBS; Gibco, USA) at 37℃ in a humidified atmosphere of 5 % CO_2_.

### Wnt3a knockout with Crispr/Cas9 system

According to human Wnt3a (NCBI: NM033131) sequence, 3 candidate sgRNAs targeting Wnt3a along with vectors (Lenti-CAS9-puro and Lenti-sgRNA-EGFP) were designed and constructed by GeneChem (Shanghai, China). sgRNA sequence was listed as follows: sgRNA-1: 5'-TGTAGCGAGGACATCGAGTT-3' (nt 570~589); sgRNA-2: 5'-CGCGCGGCGATGGCCCCACT-3' (nt 99~118); and sgRNA-3:5'-CT CTGGGCAGCTACCCGTC-3' (nt 154~173). For the transfection, briefly, 5×10^4^ HepG2 cells at 50 % confluency in 6-well plates were transfected with Lenti-CAS9-puro vector for 48 h, and then screened by puromycin. Following harvest, Lenti- sgRNA-EGFP with different sgRNAs against Wnt3a was transfected into Cas9-transfected cells. Then the transfection efficacy was analyzed under a fluorescence microscope (Olympus, Japan), with sg-RNA-CON244 as a negative control (NC) for study.

### SURVEYOR assay

The SURVEYOR assay was performed using SURVEYOR Mutation Detection Kits (IDT, USA) according to the manufacturer's instructions, and primers are shown in **Table 1**. In brief, DNA of samples with sgRNAs was harvested for PCR and SURVEYOR nuclease digestion. The PCR amplification was performed with primers, 2 × Taq Plus Master Mix, 30 ng of genomic DNA, and ddH_2_O. After the PCR amplification, the mixtures of the amplified PCR products reacted with Detecase Buffer, Detecase, and ddH_2_O at 45 ℃ for 20 min. Subsequently, the DNA fragments were electrophoresised on 2 % agarose gel at 105 V for 45 min.

### Cell cycle analysis

Cells in the sgRNA2 or NC group were collected by trypsin and washed with pre-cold PBS. Following fixed in 70 % ethanol for 12 h, cells were resuspended in 50 μg/mL RNase A solution and incubated for 30 min at 37 ℃. Then samples were incubated with 10 μg/mL propidium iodide (Solarbio, China) in the dark for 15 min. After that, cells were detected by a Flow Cytometer (BD, USA). Data was analyzed by the Modfit software.

### MTT assay

Cell proliferation was determined by MTT (thiazolyl blue tetrazolium bromide) assay. Cells (1×10^3^) in sgRNA or NC group were seeded into 96-well plates. Following incubated for 12 h, cells were treated with MTT solution at 0 h, 24 h, 48 h, 72 h, and 96 h. After removing the suspension, 90 μL of dimethyl sulfoxide (DMSO) was added to solubilize the formazan. The plate was finally read by a micro-plate reader (Bio-Rad, USA) at a wavelength of 490 nm.

### Colony formation assay

Cells in the sgRNA2 or NC group were plated in 6-well plates (500 cells/well) and incubated in complete medium for two weeks. Following fixed by 4 % paraform- aldehyde, cells were stained with 0.1 % crystal violet. Visible stained colonies were counted under a microscope (Olympus, Japan).

### Western blotting

Following extraction of total protein and sodium dodecyl sulfate-polyacrylamide gel electrophoresis (SDS-PAGE), samples were transferred onto polyvinylidene fluoride (PVDF) membranes (Bio-Rad, USA). After blocked for 4 h, the samples were incubated with rabbit anti-Wnt3a, anti-β-catenin, or anti-β-actin antibodies (1:1000, Abcam, USA) overnight at 4℃. After washing by TBST (Tris-Buffered Saline with Tween 20) solution, membranes were incubated with horseradish peroxidase (HRP)-conjugated goat anti-rabbit IgG antibodies (Univ-bio, China) at room temperature for 2 h. Subsequently, samples were visualized by electrochemiluminescence (ECL) solution (Millipore, USA).

### Immunohistochemistry (IHC)

Liver IHC assay was performed according to previously described^16^. Briefly, paraffin-embedded sections of tissues were deparaffinized in xylene, rehydrated by ethanol. Following heat-induced epitope retrieval in 0.01 Mol citrate buffer at pH 6.0 for 15 min and washed with PBS, the samples were blocked with 5 % BSA for 2 h. Then the sections were incubated with primary Wnt3a, Wnt5a or Ki67 antibodies (1:100, Abcam, USA) overnight at 4 ℃. After incubated with secondary antibodies (Univ-Bio., China) for 1 h at room temperature, sections were stained with 3′, 3-diaminobenzidinetetrahydro chloride (DAB, Sigma, USA) and counterstained with hematoxylin.

### Enzyme-linked immunosorbent assay (ELISA)

Wnt3a levels in sera and liver tissues were quantitatively detected according to manufacturer's instructions of human or rat Wnt3a ELISA Kit (Cloud Clone Corp., China). In brief, 100 µL of sample dilution and standards were added to indicated wells and incubated for 1 h at room temperature. Following washing, HRP-conjugated antibody was added and incubated for 1 h at room temperature. After washing again, each well was incubated with 100 µL of 3, 3', 5, 5'-tetramethylbenzidine (TMB) substrate solution for 30 min away from light and stopped with 90 µL of stop solution. Then the absorbance was measured in a microplate reader at 450 nm. Experiments were conducted in triplicate.

### Xenograft model

BALB female mice (about 4-6 weeks age) were provided by Animal Center of Nantong University and maintained under specific-pathogen-free conditions. HepG2 cells (5×10^6^) transfected with sgRNA2 or NC were suspended in 100 µL DMEM with Matrigel (BD Biosciences, USA), and then subcutaneously inoculated on the right flank of mice. Tumor volumes were monitored every 4 days after cells injection and calculated according to the following formula: volume = [length × (width)2)/2]. Mice were sacrificed at the 24^th^ day, and tumor tissues were weighed and detected for Wnt3a and Ki67 (Abcam, USA) with IHC assay. The study protocol was approved by the Committee on the Ethics of Animal Experiments of the Nantong University.

### Statistical analysis

Statistical analysis was performed using SPSS 18.0 software. Data were shown as mean ± standard deviation (SD). Receiver operating characteristic (ROC) curves were calculated to define the diagnostic value of Wnt3a and AFP simultaneous detection. Comparison between groups was analyzed by t student test or Chi-squared test. A *P* value less than 0.05 was considered statistically significant.

## Results

### Circulating Wnt3a expressions in chronic liver diseases

**Figure 1:**
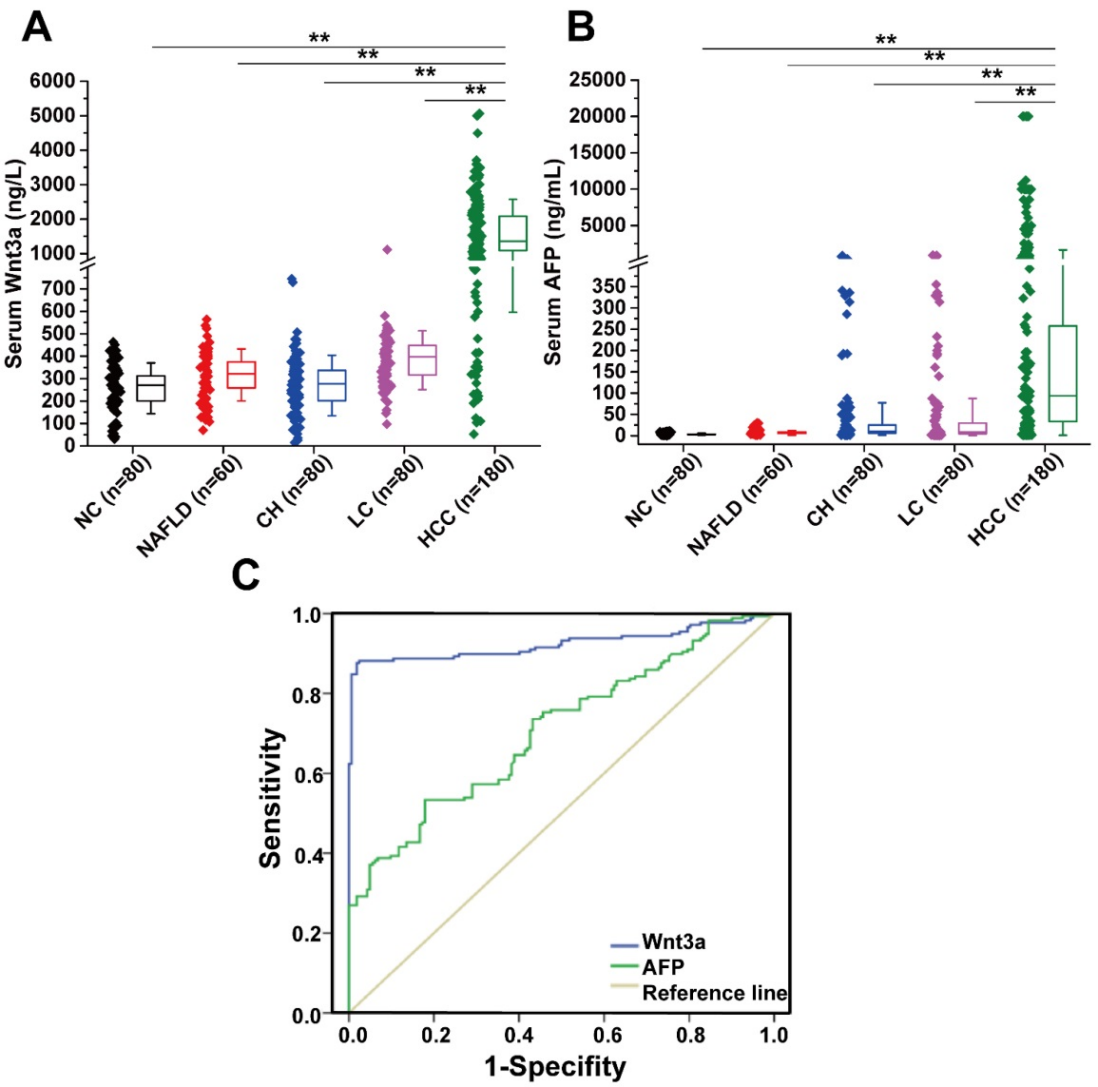
** Diagnostic value and expression features of Wnt3a for HCC. (A)** Serum Wnt3a levels were quantitatively detected in a cohort with HCC (n = 180), LC (n = 80), CH (n = 80), NAFLD (n = 60), and NC (n = 80) by an enzyme-linked immunosorbent assay, respectively. **(B)** AFP values in patients with HCC, LC, CH, NAFLD, and NC. **(C)** ROC curves of Wnt3a and AFP for HCC diagnosis. AFP, α-fetoprotein; HCC, hepatocellular carcinoma; LC, liver cirrhosis; CH, chronic hepatitis; NAFLD, nonalcohol fatty liver disease; NC, normal control. Wnt3a, Wingless-type MMTV integration site family member 3a. **, *P* < 0.01.

The levels of circulating Wnt3a expression in a cohort of 400 patients with liver diseases were detected and the comparison with AFP levels are shown in Table 2 and Figure 1. The mean Wnt3a concentration in the HCC group was significantly higher (*P*<0.001) than any group of patients with benign chronic liver diseases, with average increasing 4.2, 5.9, 5.1, and 6.2 folds than that in the LC, CH, NAFLD, and NC group, respectively. The cutoff value was set 478.0 ng/L (mean±1.96 SD) as the upper limit, and the incidence of serum Wnt3a was 87.8 % (158/180), which was more sensitive than that of AFP (>20 ng/mL, 67.2%, 121/180) in HCC patients (Fig.1B). Besides, lower positive ratio was also observed in cases with benign chronic liver diseases. Notably, the incidence of serum Wnt3a was 81.4% in HCC patients with AFP level less than 20 μg/L (48/59), 92.6% in the cases with tumor size less than 3.0 cm (75/81), and up to 93.9% when Wnt3a plus AFP combination.

### Clinicopathological features of Wnt3a expression in HCC

The clinicopathological features of abnormal Wnt3a expression in HCC are shown in **Table 3**. The up-regulated levels of Wnt3a expression in sera of HCC patients was closely associated with HBV infection (*P*<0.001), lymph node metastasis (*P*=0.016), differentiation degree (*P*=0.001), TNM staging (*P*=0.003), Child-pugh classification (*P*<0.001), and tumor recurrence (*P*=0.014). However, no significantly difference (*P*>0.05) was found between Wnt3a and HCC patients' age, gender, tumor size, AFP level, liver cirrhosis, or gross classification.

### Diagnostic value of Wnt3a for HCC

According to the ROC curve (**Fig. 1C**), the area under curve (AUC) of Wnt3a was 0.924 (CI: 0.892-0.957, P<0.001), and that of AFP was 0.707 (CI: 0.653-0.761, P<0.001). Comparative analysis of Wnt3a with other biomarkers such as routine AFP, hepatoma-specific gamma-glutaml transferase (HS-GGT)^7^, and GPC-3^8^ is shown in Table 4. Clinical evaluation of serum Wnt3a for HCC diagnosis, similar to HS-GGT, was a promising marker with higher sensitivity, specificity, accuracy, positive predictive value (PPV), and negative predictive value (NPV).

### Wnt3a expression in HCC tissues

**Figure 2:**
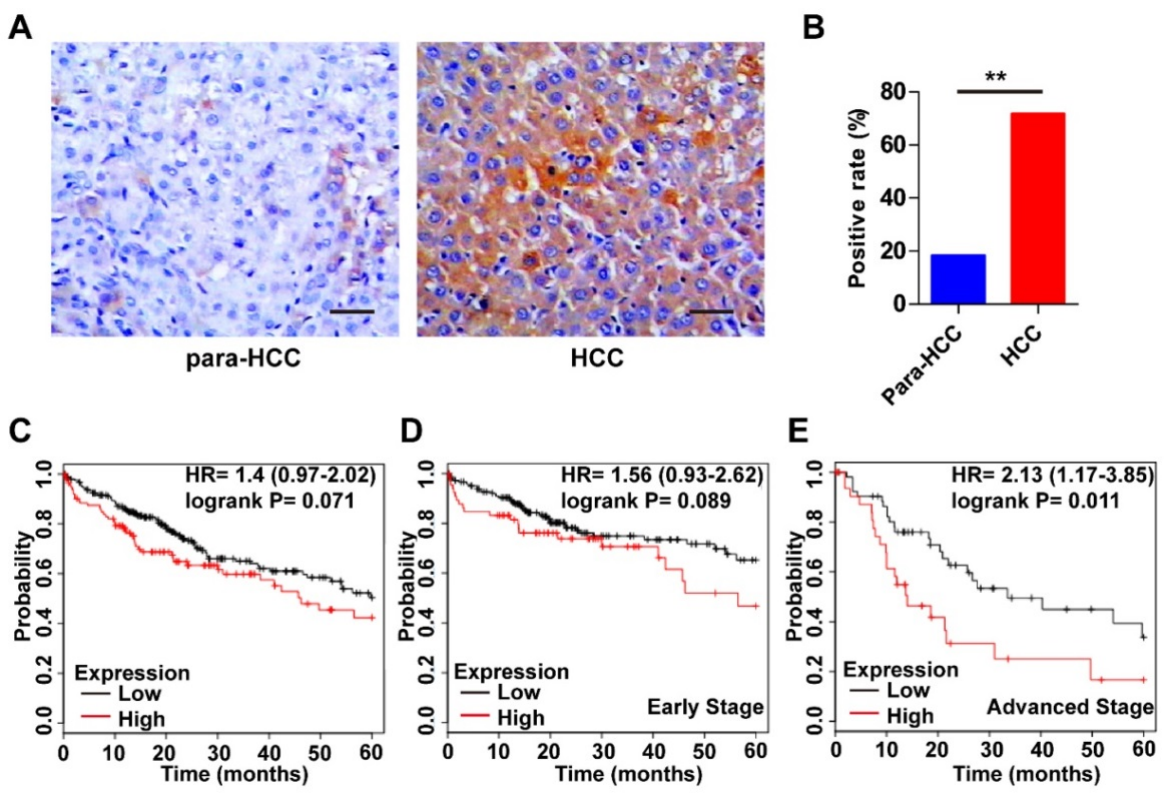
** Expression features and prognostic roles of Wnt3a in HCC tissues. (A)** Immunohistochemistry of Wnt3a in HCC and para-cancerous tissues. **(B)** The positive rate of Wnt3a in HCC and para-cancerous tissues according to immunochemical staining scores. **(C)** Kaplan-Meier curves according to high or low expression of Wnt3a in HCC patients from TCGA cohort. **(D)** Kaplan-Meier curves according to high or low expression of Wnt3a in HCC patients at early stages from TCGA cohort. **(E)** Kaplan-Meier curves according to high or low expression of Wnt3a in HCC patients at advanced stages from TCGA cohort. Wnt3a, Wingless-type MMTV integration site family member 3a; Para, para-cancerous. Bar scale, 20µm. **, *P* < 0.01.

The expression features of Wnt3a in HCC and its prognostic value are shown in Figure 2. As shown in Fig. 2A, the intensity of Wnt3a staining in HCC tissues was stronger than that in the para-cancerous tissues. Semi-quantitive analysis showed that the positive rate of hepatic Wnt3a in HCC group (71.67%, 43/60) was significantly higher (*χ*^2^ =34.478, *P*<0.001) than that in the para-cancerous group (18.33%,11/60; Fig. 2B). In addition, we also evaluated the prognostic role of Wnt3a for HCC in TCGA cohort. Though it was not statistically significant in the integral cohort or the cases at early stages, the patients with high Wnt3a expression had an obviously shorter survival (Fig. 2C&D). Notably, for the cases at advanced stages, Wnt3a expression could act as a novel prognostic factor for the survival of HCC patients (HR=2.13, *P*=0.011, Fig. 2E).

### Dynamic Wnt3a expression in hepatocarcinogenesis

**Figure 3:**
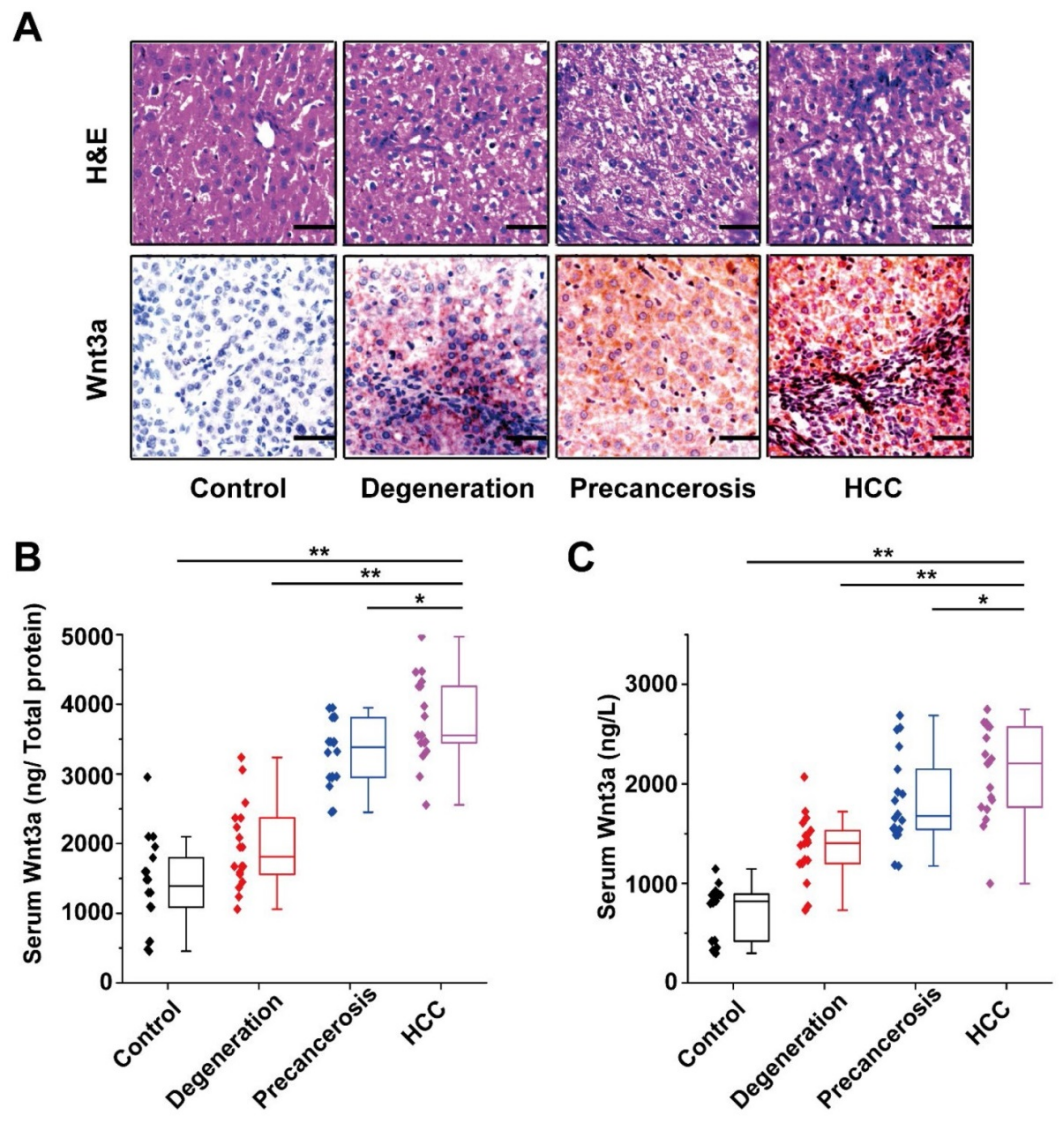
** Dynamic alteration of Wnt3a expression in hepatocarcinogenesis.** The rat dynamic model in hepatocarcinogenesis was made according to previous method and divided into control, degeneration, pre-cancerosis, and HCC groups based on liver histopathological examination with hematoxylin & eosin (H&E) staining. **(A)** The H&E staining of representative livers (upper) and the Wnt3a immunohistochemistry (below) of corresponding to livers. **(B)** The quantitative analysis of Wnt3a specific concentration (ng/μg total protein) in rat liver tissues at different stages. **(C)** The circulating Wnt3a levels in corresponding rats. Wnt3a, Wingless-type MMTV integration site family member 3a. Bar scale, 20µm. *, *P*< 0.05; **, *P*< 0.01.

The Wnt3a expression in rat hepatocarcinogenesis model is shown in **Figure 3**. According to the H&E staining, the rats were divided into four groups: control, degeneration, pre-cancerosis, and HCC. Then, IHC staining of the tissues from groups above demonstrated that Wnt3a expression significantly increased during the malignant transformation of hepatocytes (Fig. 3A). Furthermore, up-regulating Wnt3a in liver suspension and serum was also determined in the HCC formation of rats by using ELISA assay (Fig. 3B&C). In view of the remarkable alteration of Wnt3a in hepatocarcinogenesis, it might play a crucial role in HCC progression.

### Knockout of Wnt3a by Crispr/Cas9 system on biological behaviors of HCC cells

**Figure 4:**
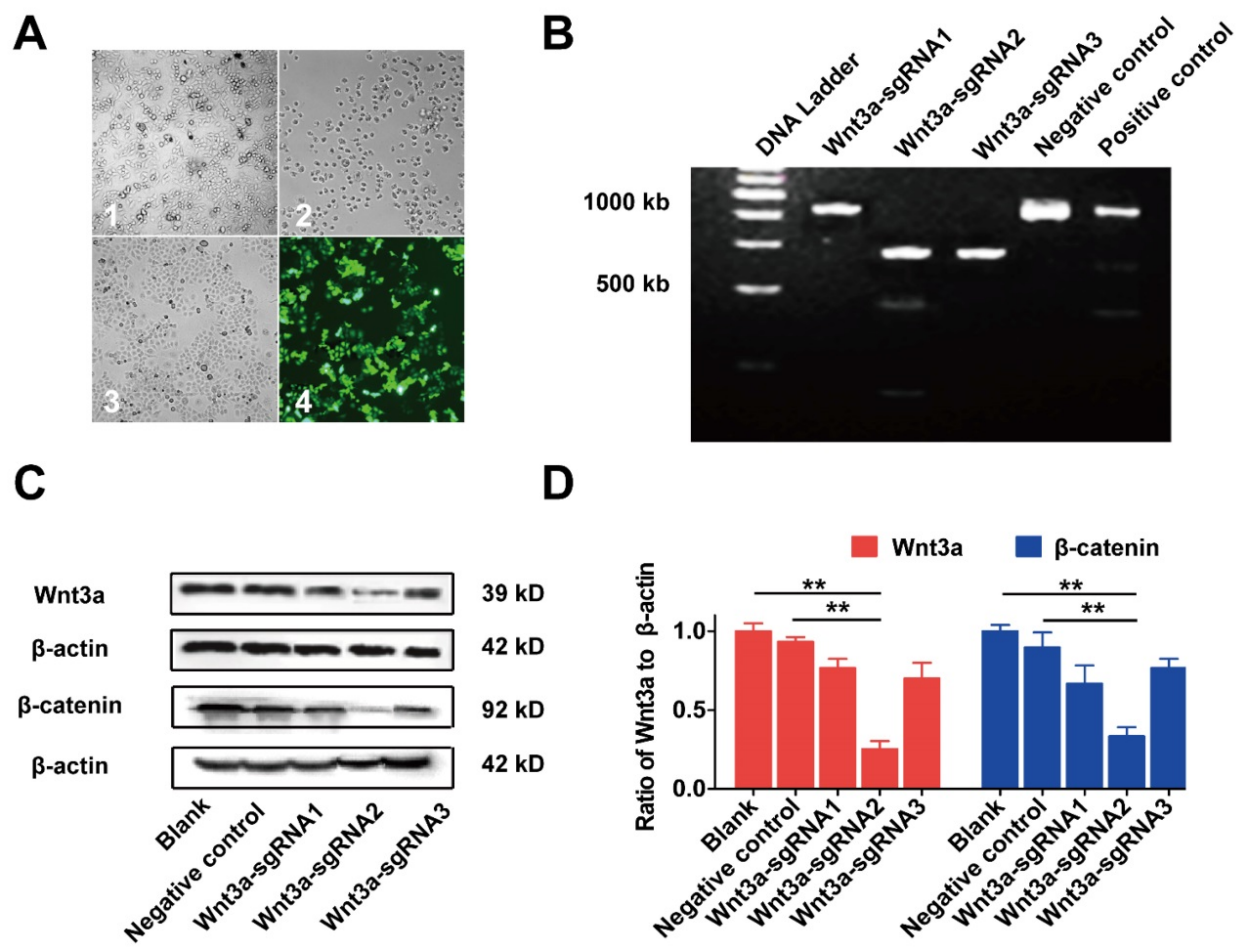
** Inhibiting Wnt3a expression of HCC cells by Crispr/Cas9 system. (A)** The lentivirus expressing Cas9 with puro-resistant Wnt3a-targeted sgRNAs and EGFP was sequentially transfected into HepG2 cells. A1, HepG2 cells infected lentivirus expressing Cas9; A2, cells were screened by puromycin; A3, cells were subsequently infected with lentivirus expressing Wnt3a-targeted sgRNAs with EGFP; A4, infection efficacy was observed by a fluorescence microscope. **(B)** The SURVEYOR gel showed that genomic modification occurred at targeted-exon loci of Wnt3a in HepG2 cells. **(C)** The protein expression of Wnt3a and its downstream gene β-catenin was detected by western blotting after infection of sgRNAs. β-actin was chosen as a loading control. **(D)** The relative intensity of bars in C. Wnt3a, Wingless-type MMTV integration site family member 3a. **, *P* < 0.01.

**Figure 5:**
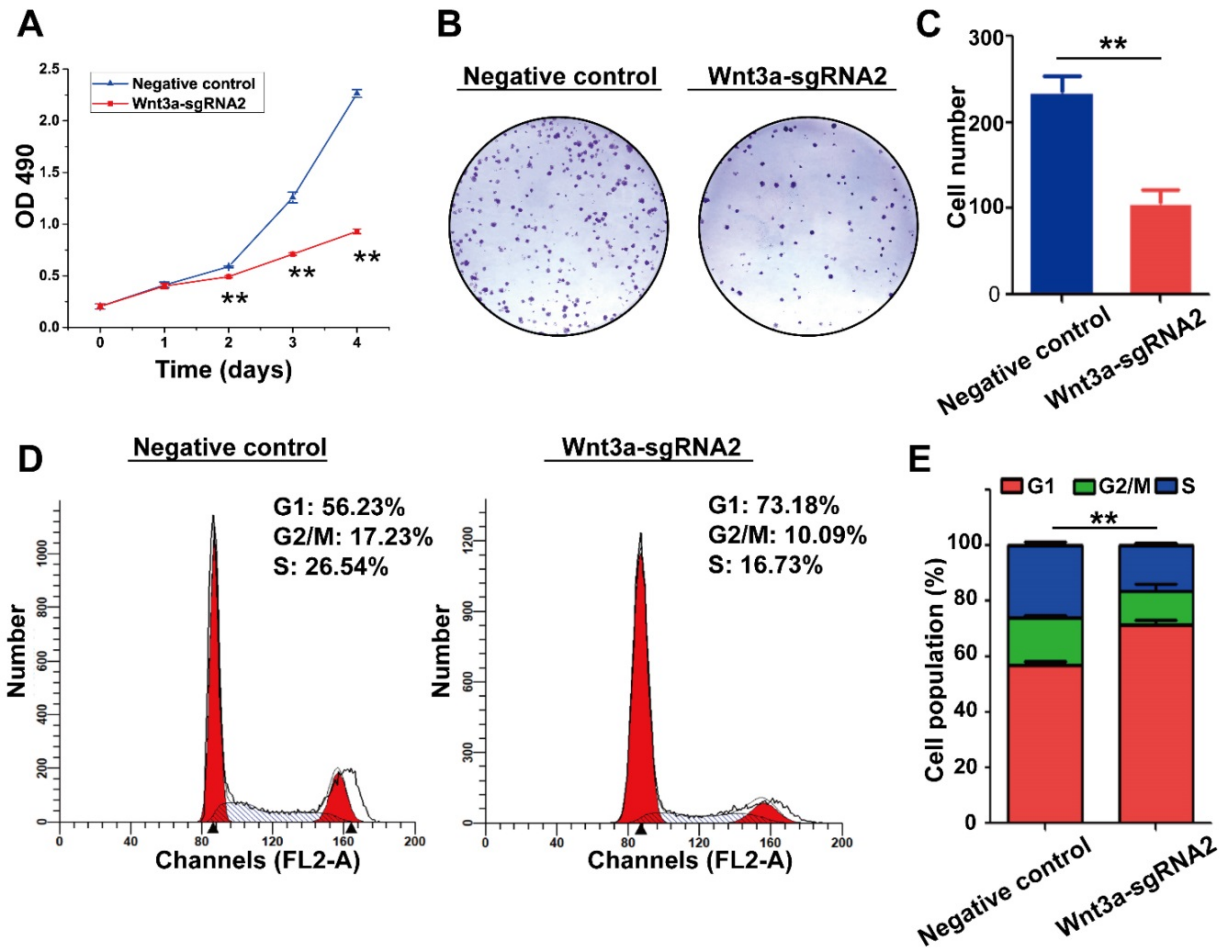
** Effects of Wnt3a Silencing on the biological behaviors of HCC cells. (A)** Cell proliferation was detected in HepG2 cells infected with sgRNA2 or NC by using MTT assay. **(B)** Colony formation of HepG2 cells infected with Wnt3a-sgRNA2 or NC. **(C)** Each bar represents the mean ± SD for three independent experiments of F. **(D)** Wnt3a-sgRNA2 or NC infected cells were analyzed for cell cycle distribution by FACS analysis. **(E)** Each bar represents the mean ± SD for three independent experiments of H. NC, negative control; Wnt3a, Wingless-type MMTV integration site family member 3a. **, *P* < 0.01.

The effects of Wnt3a silencing by Crispr/Cas9 on biological behaviors of HCC cells are shown in Figure 4 and Figure 5. Initially, HepG2 cells were infected with the lentivirus expressing Cas9 with puro-resistance (Fig. 4A1&2). Subsequently, lentivirus expressing different Wnt3a- targeted sgRNAs with EGFP was infected into puromycin-screened cells (Fig. 4A3 & 4). As shown in Fig. 4B, the SURVEYOR assay showed that sgRNA was capable of binding to the expected Wnt3a regions and facilitated the excision of genomic strands via the Cas9 enzyme. Furthermore, the protein expression of Wnt3a and its downstream gene β-catenin significantly attenuated after infection of Wnt3a-sgRNA2 (Fig. 4C&D), indicating that sgRNA2 was the most efficient sgRNA for knockout of Wnt3a. As shown in Fig. 5A, the proliferation rate was significantly inhibited in HepG2 cells infected with Wnt3a-sgRNA2. Besides, the colony number of HepG2 cells also obviously reduced after Wnt3a knockout (236.00 ± 17.78 vs. 106.67 ± 15.57; *t* = 9.480, *P* = 0.001; Fig. 4 B&C). Furthermore, flow cytometry analysis showed that Wnt3a gene knockout by sgRNA2 also led to cell cycle arrest in G1 phase in HepG2 cells (*P* < 0.01; Fig. 4 D&E).

### Effects of Wnt3a on HCC xenograft growths* in vivo*

**Figure 6:**
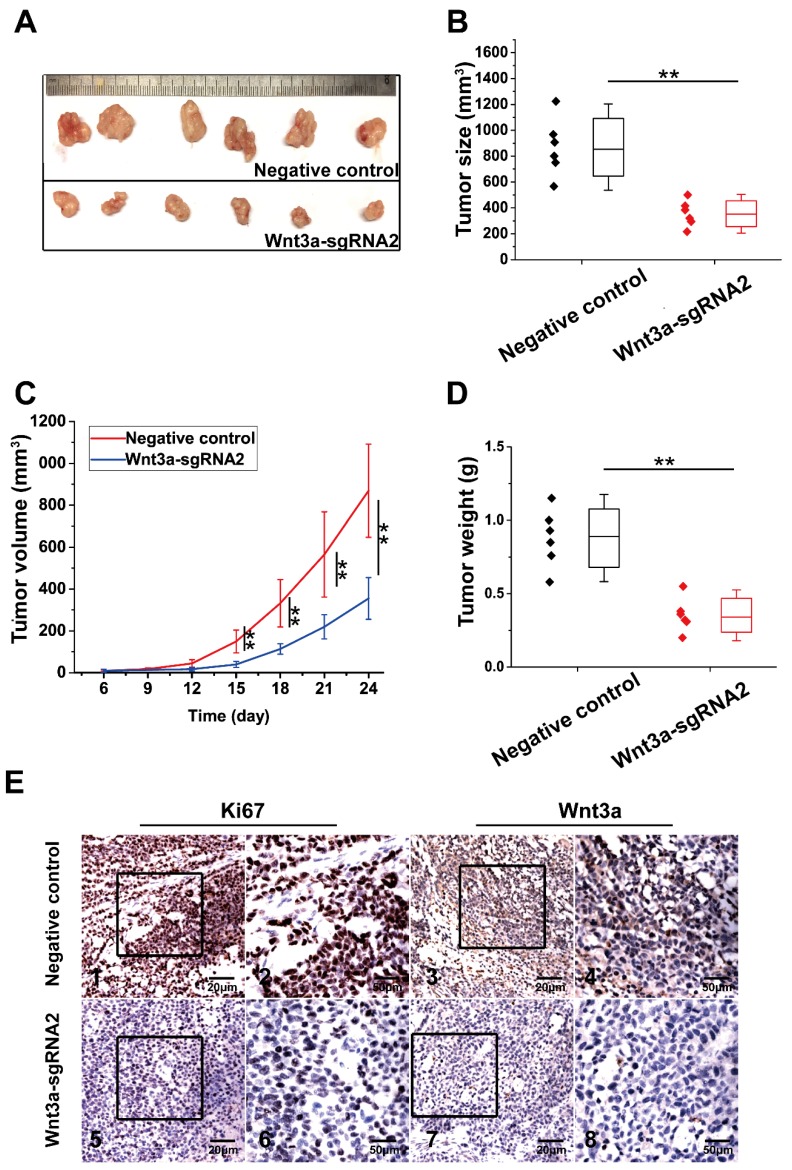
** Effects of Wnt3a silencing on tumor growth *in vivo*. (A)** HepG2 cells infected with NC or Wnt3a-sgRNA2 were subcutaneously injected into mice, and tumors were resected at 24th day after injection; **(B)** The final volume of xenograft tumors in NC or Wnt3a-sgRNA group. **(C)** The growth curves of xenograft tumors in NC or Wnt3a-sgRNA group; **(D)** The weight of xenograft tumors in NC or Wnt3a-sgRNA group; **(E)** The IHC staining of Ki67 (E1, E2, E5, and E6) or Wnt3a (E3, E4, E7, and E8) in tumor tissues of NC or Wnt3a-sgRNA group. Wnt3a, Wingless-type MMTV integration site family member 3a. **,* P* < 0.01.

The effect of Wnt3a knockout on xenograft growth is presented in **Figure 6**. HepG2 cells of NC group and Wnt3a-sgRNA2 group were subcutaneously injected into mice, and tumors were resected at the 24th day after injection (Fig. 6A). As shown in Fig. 6B & D, tumors of the Wnt3a-sgRNA2 group had smaller volume (355.00 ± 99.85 mm^3^ vs. 869.42 ± 222.46 mm^3^; *t* = 5.168, *P* < 0.001) and lighter weight (0.35 ± 0.11g vs. 0.88 ± 0.20g; *t* = 5.628, *P* < 0.001) than the NC group. Besides, the growth rate of tumors in the Wnt3a-sgRNA2 group was much slower than that in the NC group (Fig. 6C). Furthermore, IHC analysis confirmed that the expressions of Wnt3a and Ki67 were significantly decreased in the xenograft tissues of the Wnt3a-sgRNA2 group with weaker staining intensity (Fig. 6E).

## Discussion

Early diagnosis and effective therapy of HCC are of the utmost importance. From a population-based cancer registry for 40 years, HCC still ranks the leading incidence among all malignancies and the poorest survival rate in the area inshore of the Yangtze River, China^2^. The Wnt/β-catenin pathway-related signaling including Wnt ligands, GSK-3β, β-catenin, and β-catenin-mediated downstream genes play crucial roles in HCC progression, including hepatocytes malignant transformation, metastasis, chemoresistance, liver cancer stem cells, and formation of epithelial-mesenchymal transition^17^. Our previous work, for the first time, revealed abnormality of oncogenic Wnt3a in the sera and cancerous tissue of HCC patients. However, its underlying mechanisms and the expression features in hepatocarcinogenesis remain to be identified. In the present study, we continued to explore dynamic expression of Wnt3a in hepatocarcinogenesis and evaluate its diagnostic and molecular-targeted value.

Wnt/β-catenin signaling pathway plays a significant role in numerous biological process 18. However, abnormal Wnt signaling was recently associated with HCC occurrence and progression, especially in canonical pathway Wnt3a activation or non-canonical pathway Wnt5a inactivation^19^. Previous work highlighted the correlations of Wnt3a levels with aggressive phenotype in HCC tissues^20^. Consistently, the current study demonstrated that Wnt3a expression in cancerous tissues was higher than that of para-cancerous tissues. In addition, survival analysis in large HCC cohort from TCGA indicated that Wnt3a could serve as a prognostic factor in HCC cases at advanced stages. Furthermore, the rat model discovered that the Wnt3a expression markedly increased in hepatocarcinogenesis, with gradually up-regulating Wnt3a at protein levels in sera and livers of rats. Dynamic expression of Wnt3a has been involved in rat liver tumorigenesis, and associated with malignant transformation of hepatocytes, indicating that Wnt3a might participated in promoting tumorigenesis and progression of HCC.

Human Wnt3a gene located on chromosome (1q42.13) has been regarded as an activator for β-catenin accumulation and the canonical Wnt signaling pathway^21^. The amino-terminal region of Wnt proteins may mediate interactions with Wnt receptors and activate them by the carboxyl terminus. As expected, the level of serum Wnt3a expression in the HCC group was significantly higher than that in any of other benign liver disease groups and healthy control, which obviously correlated with malignant features. Besides, its superiority of Wnt3a similar to HS-GGT, over AFP or GPC-3 in HCC diagnosis was also observed in the current study. The current data, consistent with the previous work, further confirmed that the abnormal oncogenic Wnt3a expression in HCC progression, suggesting Wnt3a as a candidate specific biomarker for HCC diagnosis and differentiation.

The Wnt signaling includes two parts: one is through canonical pathway characterized by the stabilization and subsequent nuclear transport of β-catenin resulting in the activation of transcriptional responses; the other is non-canonical pathway with more diverse and several different signaling modes that regulate cell biological behaviors. Accumulating evidence indicates that viral proteins of HBV (HBx, HBsAg) or HCV (Core or NS) as pathogenic factors provoke activation of Wnt/β-catenin pathway in hepatocarcinogenesis^22^. Interaction of Wnt signals with HBV or HCV genome linked β-catenin phosphorylation and abnormalities in the E-cadherin-catenin unit function lead to loss of intercellular junctions, progression in development of cirrhosis and HCC. Oncogenic Wnt3a plays a crucial role in cell proliferation and metastasis, particularly in progression and mediated-oncogenesis involving signaling pathways. Along these lines, the Wnt pathway has been identified as contributing to the development and progression of HCC^23^.

Effective treatment of HCC is still a challenging problem worldwide. Therefore, developing novel molecule-targeted therapies may provide greater chance for effective therapies or overcome resistance to sorafenib^24^. Wnt3a is an important regulator of human HCC cell line growth, which induces activation of the canonical Wnt pathway after binding with SULF2 and GPC-3. Targeting oncogenic GPC-3 of Wnt upstream inhibited the proliferation of HCC cells^25^. Our study found that knockout Wnt3a reduced the proliferation and colony formation of HepG2 cells, with the cell cycle arrest in G1 phase *in vitro*. Besides, silencing Wnt3a significantly inhibited the xenograft growth, along with the down-regulation of proliferation marker Ki67, suggesting that Wnt3a could be a promising and effective target for HCC therapy.

In conclusion, circulating oncogenic Wnt3a as a candidate marker was further confirmed in a cohort of patients with HCC or chronic liver diseases. High Wnt3a expression could serve as a prognostic factor in HCC patients at advanced stages. Furthermore, the dynamic alteration of Wnt3a was for first time observed in a hepatocarcinogenesis model. Besides, silencing Wnt3a inhibited cancer cells proliferation* in vitro* and significantly suppress tumor growth *in vivo*. Taken together, Wnt3a might be a promising specific biomarker or an effective target for HCC therapy. However, the current works also have some limitations, and further studies should clarify its exact regulatory mechanisms, functions and application.

## Acknowledge

This study was supported by grants from National Natural Science Foundation (81673241, 81702419), Projects of Jiangsu Medical Science (BE2016698) & Graduate innovation (KYCX17_1934), Nantong Health and Family Planning Commission (WQ2016083), and International Science & Technology Cooperation Program (2013DFA32150) of China.

## Figures and Tables

**Table 1:** PCR primers for the SURVEYOR assay

Primer	Sequence (5'~3')	Amplified fragment (bp)	After enzyme digestion (bp)	
			fragment 1	fragment 2
SgRNA1	F: TCTCTCTACAGCTACCAGGG	808	177	631
	R:GGCTCACGAAGCTTTACACG	808	177	631
SgRNA2	F:ACCAGCACGTCCTCAGACAC	597	424	173
	R:CAGGTCCAAAGTGGCCGTCAA	597	424	173
SgRNA3	F: ACCAGCACGTCCTCAGACAC	597	424	173
	R:CAGGTCCAAAGTGGCCGTCAA	597	424	173

**Table 2:** Serum Wnt3a and AFP levels in patients with liver diseases

Group	n	Mean ± SD	t value*	P value*
**Wnt3a**		**ng/L**		
Hepatocellular carcinoma	180	1599.10 ± 985.81		
Liver cirrhosis	80	382.57 ±131.41	16.148	<0.001
Chronic hepatitis	80	269.24 ±135.23	17.632	<0.001
Nonalcohol fatty liver diseases	60	316.45±115.88	17.014	<0.001
Normal controls	80	256.70 ±112.96	17.908	<0.001
**AFP**		**μg/L**		
Hepatocellular carcinoma	180	2147.11 ± 4477.21		
Liver cirrhosis	80	104.42 ± 212.87	6.072	<0.001
Chronic hepatitis	80	77.39 ± 165.49	6.158	<0.001
Nonalcohol fatty liver diseases	60	8.49 ± 6.60	6.373	<0.001
Normal controls	80	3.91 ± 2.48	6.387	<0.001

*Compared with the HCC group. SD, the standard deviation; Wnt3a, Wingless-type MMTV integration site family member 3a; AFP, α-fetoprotein.

**Table 3:** Clinicopathological characteristics of serological Wnt3a levels in 180 patients with HCC

Group	n	Mean ± SD (ng/L)	t value	P value		Positive, n (%)	χ^2^ value	P value
Age (years)								
≤60	103	1613.7±1011.6	0.443	0.658		92(89.3)	0.534	0.465
>60	77					66(85.7)		
Gender								
Male	135	1603.0±1024.5	0.414	0.680		118(87.4)	0.069	0.793
Female	45	1532.5±882.0				40(88.9)		
AFP (ng/mL)								
≤ 20	59	1416.9±871.7	1.603	0.111		48(81.4)	3.374	0.066
> 20	121	1667.5±1034.7				110(90.9)		
Cirrhosis								
With	136	1514.0±871.0	1.417	0.162		119(87.5)	0.04	0.841
Without	44	1806.0±1273.9				39(88.6)		
Tumor size								
≤ 3.0 cm	81	1559.0±931.0	0.323	0.747		75(92.6)	1.166	0.245
> 3.0 cm	99	1607.0±1038.0				83(83.4)		
HBsAg								
Positive	121	1823.7±1010.7	5.005	<0.001		113(93.4)	10.832	0.001
Negative	59	1146.1±758.3				45(76.3)		
Lymph node metastasis								
With	84	1858.2±1049.3	3.575	<0.001		79(94.1)	5.771	0.016
Without	96	1346.6±870.1				79(82.3)		
Differentiation								
Well	57	1236.7±809.1	3.308	0.001		43(75.4)	11.838	0.001
Moderate & Poor	123	1746.9±1025.2				115(93.5)		
Gross classification								
Multifocal	77	1662.2±1035.8	0.901	0.369		68(88.3)	0.036	0.850
Unifocal	103	1527.9±953.3				90(87.4)		
TNM staging								
I & II	82	1351.3±979.9	2.968		0.003	67(82.1)	5.173	0.023
III & IV	98	1781.2±957.9				91(92.2)		
Child classification								
A	98	1253.0±797.8	5.163	<0.001		78(79.6)	13.437	<0.001
B&C	82	1982.6±961.7				80(97.6)		
Recurrence								
With	47	2511.9±1153.5	7.045	<0.001		46(97.9)	6.042	0.014
Without	133	1258.0±669.8				112(84.2)		
								

Wnt3a, Wingless-type MMTV integration site family member 3a; AFP, α-fetoprotein; TNM, tumor-node-metastasis.

**Table 4:** Comparative analysis of circulating Wnt3a, AFP, HS-GGT, and GPC-3 detection in diagnosis of hepatocellular carcinoma

	Wnt3a (>478.10ng/L)	AFP (>20 ng/mL)	HS-GGT11 (>5.5 U/L)	GPC-312 (Positive)	Wnt3a +AFP
Sensitivity (%)	87.78	67.22	85.70	52.84	93.89
Specificity (%)	89.38	58.13	97.24	99.58	52.50
Accuracy (%)	88.53	62.94	96.20	83.57	74.41
PPV (%)	90.29	64.36	89.70	98.48	68.98
NPV (%)	86.67	61.18	92.23	80.20	88.42

PPV: positive predictive value; NPV: negative predictive value; Wnt3a +AFP: combining detection of Wnt3a and AFP; Wnt3a, Wingless-type MMTV integration site family member 3a; AFP, α-fetoprotein; HS-GGT, hepatoma specific γ-glutamyl transferase 7, and GPC-3, oncofetal antigen glypican-3^4^.
